# Implication of the PTN/RPTPβ/ζ Signaling Pathway in Acute Ethanol Neuroinflammation in Both Sexes: A Comparative Study with LPS

**DOI:** 10.3390/biomedicines11051318

**Published:** 2023-04-28

**Authors:** María Rodríguez-Zapata, Milagros Galán-Llario, Héctor Cañeque-Rufo, Julio Sevillano, María Gracia Sánchez-Alonso, José M. Zapico, Marcel Ferrer-Alcón, María Uribarri, Beatriz de Pascual-Teresa, María del Pilar Ramos-Álvarez, Gonzalo Herradón, Carmen Pérez-García, Esther Gramage

**Affiliations:** 1 Departamento de Ciencias Farmacéuticas y de la Salud, Facultad de Farmacia, Universidad San Pablo-CEU, CEU Universities, Urbanización Montepríncipe, Boadilla del Monte, 28660 Madrid, Spain; 2 Departamento de Química y Bioquímica, Facultad de Farmacia, Universidad San Pablo-CEU, CEU Universities, Urbanización Montepríncipe, Boadilla del Monte, 28660 Madrid, Spain; 3 BRAINco Biopharma, S.L., Bizkaia Technology Park, Zamudio, 48170 Vizcaya, Spain; 4 Instituto de Estudios de las Adicciones, Universidad San Pablo-CEU, CEU Universities, Urbanización Montepríncipe, Boadilla del Monte, 28660 Madrid, Spain

**Keywords:** pleiotrophin, Midkine, PTPRZ1, neuroinflammation, alcohol, adolescence, LPS

## Abstract

Binge drinking during adolescence increases the risk of alcohol use disorder, possibly by involving alterations of neuroimmune responses. Pleiotrophin (PTN) is a cytokine that inhibits Receptor Protein Tyrosine Phosphatase (RPTP) β/ζ. PTN and MY10, an RPTPβ/ζ pharmacological inhibitor, modulate ethanol behavioral and microglial responses in adult mice. Now, to study the contribution of endogenous PTN and the implication of its receptor RPTPβ/ζ in the neuroinflammatory response in the prefrontal cortex (PFC) after acute ethanol exposure in adolescence, we used MY10 (60 mg/kg) treatment and mice with transgenic PTN overexpression in the brain. Cytokine levels by X-MAP technology and gene expression of neuroinflammatory markers were determined 18 h after ethanol administration (6 g/kg) and compared with determinations performed 18 h after LPS administration (5 g/kg). Our data indicate that *Ccl2*, *Il6,* and *Tnfa* play important roles as mediators of PTN modulatory actions on the effects of ethanol in the adolescent PFC. The data suggest PTN and RPTPβ/ζ as targets to differentially modulate neuroinflammation in different contexts. In this regard, we identified for the first time important sex differences that affect the ability of the PTN/RPTPβ/ζ signaling pathway to modulate ethanol and LPS actions in the adolescent mouse brain.

## 1. Introduction

Binge drinking is a common practice worldwide and contributes to an increased risk of developing alcohol use disorder (AUD). In 2020, 61.1 million people were binge alcohol (ethanol) users [1]. Specifically, in Spain, up to 70.5% of adolescents reported ethanol consumption during the last 12 months. It is important to note that gender differences regarding the prevalence of consumption and the prevalence of binge drinking have been reported, both higher in adolescent girls than in boys [2]. Adolescence is a critical period for consumption because the brain is more vulnerable to the damaging effects of ethanol [3,4]. Evidence obtained from experimental animals has shown that binge-like ethanol exposure during adolescence impairs prefrontal cortex (PFC) maturation and hippocampal neurogenesis and induces synaptic and myelin disarrangements [5,6].

Different studies suggest the role of neuroinflammation in the pathophysiology of excessive ethanol consumption [7,8]. However, the molecular mechanisms of ethanol actions in the adolescent brain remain elusive. It is important to note that females seem to be remarkably vulnerable to ethanol damage [5,9,10], and more susceptible than males to the neurodevelopmental dysfunction associated with heavy ethanol use in adolescence [11]. 

Ethanol activates the neuroimmune system and the signaling pathway of toll-like receptor 4 (TLR4) in glial cells and contributes to the release of cytokines and proinflammatory mediators [5,12]. Among others, Interleukin 6 (IL6), Interleukin 1β (IL1β), and tumor necrosis factor α (TNFα) are key elements of the proinflammatory response to ethanol [13,14,15]. In addition, monocyte chemoattractant protein 1 (MCP1), one of the most potent microglia chemokines, which is produced by many other central nervous system (CNS) cell types, has emerged as an important factor that regulates neuroinflammation in different contexts [16,17]. Acute ethanol treatment increases brain mRNA levels of *Il6*, *Il1β*, *Tnfa*, and *Ccl2* (MCP1) proinflammatory genes in mice. However, these changes together with microglial alterations seem to be region-specific and sensitive to timing [9,14,15,18]. 

The genetic elimination of TLR4, whose most relevant ligand is lipopolysaccharide (LPS), modulates neuroinflammation and other ethanol-deleterious effects in the mouse brain [5,19]. In addition, during acute ethanol intoxication, an association between elevated cytokine/chemokine levels and the upregulation of TLR4 mRNA levels in total blood was found exclusively in females, pointing out important sex differences in the immune response to ethanol [11]. Moreover, recent studies have shown an increase in blood LPS levels after ethanol binge drinking, suggesting a connection between the release of the endotoxin through systemic circulation and the neuroinflammation induced by the drug [20]. However, as ethanol itself can trigger the TLR4 response in microglia [21,22], further studies are needed to clarify the specific contribution of ethanol and the release of LPS in this neuroinflammatory process.

Pleiotrophin (PTN) is a cytokine that plays a key role in CNS repair and the differentiation of neurons. It is part of the *Ptn*/*Mdk* developmental gene family, sharing over 50% identity in amino acid sequence with Midkine (MK) and hence many functions [23]. Interestingly, PTN is upregulated in the mouse brain after the administration of ethanol [24,25] and MK expression is higher in the frontal cortex of human alcoholics [26]. In fact, both cytokines are involved in the modulation of the neuroimmune response and neuroinflammation induced by different drugs of abuse [27]. In addition, we demonstrated that in *Ptn* knockout mice (*Ptn*^−/−^), the conditioning effects of ethanol are significantly increased, whereas the transgenic overexpression of *Ptn* in the mouse brain (*Ptn*-Tg) blocks the rewarding effects of this drug [25]. This evidence suggests that PTN/MK may regulate ethanol effects through the modulation of neuroinflammation.

PTN and MK exert their actions through different receptors [27]. One of them, the Receptor Protein Tyrosine Phosphatase β/ζ (RPTPβ/ζ, also known as PTPRZ1) is mainly expressed in the CNS in important areas for ethanol effects, such as the PFC, amygdala, or hippocampus [28]. Accordingly, RPTPβ/ζ is one of the most relevant receptors in the modulation of PTN effects in neuroinflammation [27]. The binding of PTN to RPTPβ/ζ induces its dimerization, and thus inactivates its phosphatase activity [29], increasing the phosphorylation levels of the RPTPβ/ζ substrates [30,31,32]. Some of them are known to play important roles in ethanol consumption such as anaplastic lymphoma kinase (ALK) [33] and Fyn kinase [34]. ALK has also been identified as a PTN receptor [35]. On the other hand, it has been proven that Fyn kinase participates in the triggered signaling pathway in the dorsal striatum during ethanol exposure. Moreover, this kinase plays an important role in the molecular mechanisms implicated in ethanol self-administration [23,36]. Interestingly, Fyn kinase activation participates in LPS signaling through TLR4 and in the consequent neuroinflammation process [37]. On the other hand, the pharmacological inhibition of ALK reduces ethanol binge-drinking behavior in rodents [33]. The specific contribution of the PTN/RPTPβ/ζ signaling pathway in the ethanol-induced neuroimmune response remains unclear.

Recently, we designed and synthesized a blood–brain barrier (BBB) permeable selective inhibitor of RPTPβ/ζ, named MY10. This compound has shown similar biological activities in vivo to PTN, the physiological inhibitory ligand of RPTPβ/ζ. MY10 interacts with the intracellular domain PD1 of the receptor and inhibits its tyrosine phosphatase activity, thus mimicking the inhibitory action of PTN [38]. The administration of MY10 in mice has been demonstrated to reduce ethanol consumption and to block ethanol-reinforcing actions [39,40,41], an effect also found in mice with *Ptn* overexpression in the brain (*Ptn*-Tg mice) [25]. Accordingly, RPTPβ/ζ inhibition by MY10 replicates PTN actions, increasing LPS-induced microglial response (Iba1 immunoreactivity after 7.5 mg/kg of LPS) in the PFC of adult mice [42,43]. Recently, we demonstrated that the pharmacological inhibition of the phosphatase activity of RPTPβ/ζ fully prevented the loss of hippocampal neurogenesis induced by acute ethanol exposure [44]. Moreover, that work suggested an important role of RPTPβ/ζ function in ethanol-induced immune responses in the hippocampus in a sex-dependent manner. Therefore, the aim of the present study is to elucidate the implication of the PTN/RPTPβ/ζ signaling pathway in ethanol-induced neuroinflammation in the PFC of adolescent male and female mice. In addition, we aimed to explore the possible similarities with the LPS-induced neuroinflammatory process. 

Our results suggest that the PTN/RPTPβ/ζ signaling pathway differently participates in the neuroinflammatory processes induced by ethanol and LPS. Moreover, possible differences in RPTPβ/ζ function in neuroinflammation in males and females are suggested.

## 2. Materials and Methods

### 2.1. Animals 

*Ptn*-Tg mice on a C57BL/6J background were generated by pronuclear injection as previously described [25]. The acceptor vector contained the regulatory regions responsible for tissue-specific expression of the Thy-1 gene, which drives neuron-specific expression of transgenes [45,46]. *Ptn*-specific overexpression in different brain areas of adult mice, including a ~2–3-fold upregulation in the prefrontal cortex (PFC) and a 20% increase in PTN protein levels in the striatum, was established by quantitative real-time polymerase chain reaction (qRT-PCR), in situ hybridization, and by Western blot [25,42,47]. 

Male and female C57BL/6J (wild type, *Ptn*^+/+^) and *Ptn*-Tg mice of 5–6 weeks of age (15–22 g) were used. Mice were housed under controlled environmental conditions (22 ± 1 °C and a 12-h light/12-h dark cycle) with free access to food and water. All the animals used in this study were maintained in accordance with the ARRIVE guidelines and with the European Union Laboratory Animal Care Rules (2010/63/EU directive), and protocols were approved by the Animal Research Committee of USP-CEU (authorization reference: PROEX 76.0/20). 

### 2.2. Treatments 

The RPTPβ/ζ inhibitor MY10 was synthesized as previously described [37]. Female and male *Ptn*^+/+^ mice were administered with 60 mg/kg MY10 or vehicle (Veh; 10% dehydrated ethanol, 20% polysorbate 80, 70% PEG-300) by oral gavage in a volume of 0.1 mL one hour before a single i.p. injection of ethanol (6 g/kg, 25% *v*/*v*), LPS (5 mg/kg, 10 mL/kg) or saline (control). MY10 treatment was based on previous studies with this compound, in which we demonstrated that this dose and route of administration reduces ethanol consumption, prevents ethanol-induced loss of hippocampal neurogenesis, and provides good bioavailability and BBB penetration in mice [38,39,41,44]. The dose of ethanol was chosen to achieve in mice Blood Ethanol Concentrations (BECs) equivalent to those found in human adolescents after binge alcohol drinking (peak blood concentration, 700–750 mg/dL) [48,49]. All animals were fully recovered at the time of sacrifice, 18 h after ethanol administration. 

To study the effect of the endogenous overexpression of PTN, *Ptn*-Tg mice were compared with *Ptn*^+/+^ with the same treatment. On the other hand, the effect of the inhibition of RPTPβ/ζ by MY10 treatment was evaluated only in *Ptn*^+/+^ mice. To minimize the number of animals used in the study and apply the 3Rs principles, the same control mice were used in both sets of experiments. For that reason, all the mice used in the study received an oral administration of the vehicle independently of the genotype (except for *Ptn*^+/+^ mice treated with MY10). 

Mice were decapitated 18 h after ethanol, LPS, or saline administration and PFC were rapidly removed and frozen in dry ice and stored at −80 °C until the procedures. To quantify protein levels of cytokines, ethanol-treated mice and their controls were used (n = 3–7/group/sex). For tissular mRNA expression analysis, ethanol- and LPS-treated mice and their controls were used (n = 5–6/group/sex). 

### 2.3. Cytokine Levels 

Approximately 5 mg of PFC were homogenized in 100 μL of homogenization buffer (0.05% Tween 20 and protease inhibitor cocktail (Thermo Fisher Scientific Inc., Waltham, MA, USA) in PBS, pH 7.2). Samples were processed as previously described [43] and the total protein content of each sample was measured using the BCA protein assay kit (Thermo Fisher Scientific Inc., Waltham, MA, USA). Levels of IL1β, IL6, MCP1, and TNFα were measured by multiplex luminometry (Beadlyte mouse multiplex cytokine detection system, MHSTCMAG-70K, Merck Millipore, Spain) according to the manufacturer’s description. 

### 2.4. Quantitative Real-Time PCR 

Total RNA from PFC was isolated using the Total RNA Isolation Kit (Nzytech, Lisbon, Portugal). First-strand cDNA was synthesized using the first-strand cDNA Synthesis Kit (Nzytech). Quantitative real-time PCR analysis was performed using the SYBR green method (Quantimix Easy kit, Biotools, Madrid, Spain) in a CFX96 Real-Time System (Bio-Rad, Hercules, CA, USA). The relative expression of each gene, in brain tissue, was normalized using *Rpl37*. The primer sequences are shown in Table 1.

### 2.5. Statistical Analysis

All statistical analyses were performed using Graphpad Prism version 8 (San Diego, CA, USA). Data are presented as mean ± standard error of the mean (SEM). 

To elucidate the role of endogenous PTN, *Ptn*-Tg mice were compared with wild type (*Ptn*^+/+^) mice, and the results were analyzed using a three-way ANOVA, considering treatment, genotype, and sex as variables. To better discern the effect of the treatment and of the genotype, when a significant interaction between treatment and genotype variables was found and the absence of a sex effect was confirmed, we performed a two-way ANOVA, excluding the sex factor. When three-way ANOVA revealed that sex and/or treatment variables were significant, it was properly represented in the graph. No further comparisons were performed in those cases. For detailed results of these statistical analyses see Supplementary Tables S2 and S4.

The implication of the RPTPβ/ζ signaling pathway was assessed through the pharmacological inhibition of the receptor with MY10. In this case, data were analyzed using a two-way ANOVA, having as variables treatment and sex (Tables S3 and S5). 

To comparatively study the expression of RPTPβ/ζ signaling pathway components in ethanol and LPS models, males and females were separately analyzed. Two-way ANOVA was used when results from *Ptn*-Tg mice were included in the analysis (considering treatment and genotype as variables), and one-way ANOVA was performed when only different treatments were tested in wild type (*Ptn*^+/+^) mice (Table S6). 

All references to statistical significance made to the three-way, two-way, and one-way ANOVA test’s individual factors or their interaction are shown in Supplementary Tables S1–S6. In the figures, only significant differences revealed by post-hoc comparisons with Bonferroni’s post-hoc test are represented.

## 3. Results

### 3.1. Ptn Overexpression Attenuates Acute Ethanol-Induced MCP1 Increment in the PFC of Male and Female Mice

**Figure 1:**
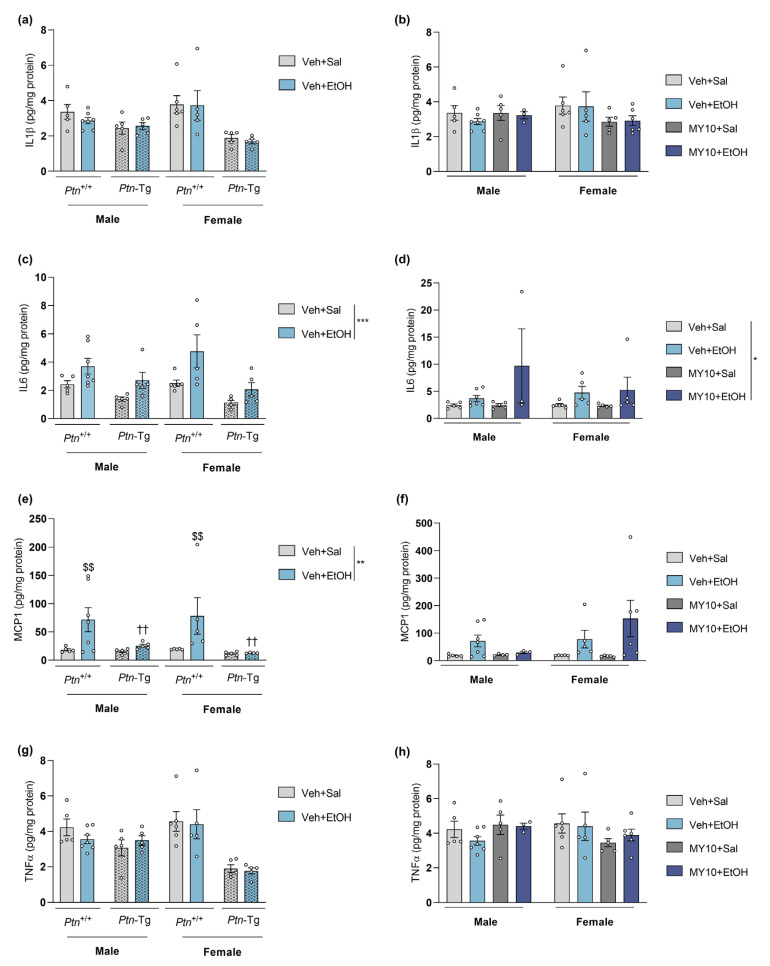
Cytokine expression in the PFC of adolescent male and female mice after acute ethanol administration. Graphs show data from a genetic *Ptn* overexpression model (*Ptn*-Tg vs. *Ptn*^+/+^ mice; **a**,**c**,**e**,**g**) and data from *Ptn*^+/+^ mice with RPTPβ/ζ inhibition by MY10 (**b**,**d**,**f**,**h**). Protein levels of (**a**,**b**) IL1β: Interleukin 1β; (**c**,**d**) IL6: Interleukin 6; (**e**,**f**) MCP1: monocyte chemoattractant protein 1 and (**g**,**h**) TNFα: tumor necrosis factor α were measured using multiplex luminometry. Three and two-way ANOVA analysis was performed, when appropriate. Data are presented as mean ± SEM (n = 3–7/group). * *p* < 0.05, ** *p* < 0.01, *** *p* < 0.001. If appropriate, only genotype and treatment factors were considered. $$ *p* < 0.01 vs. vehicle-saline within the same genotype, excluding sex factor. †† *p* < 0.01 vs. *Ptn*^+/+^ vehicle-ethanol, excluding sex factor.

To explore the implication of the overexpression of endogenous PTN (*Ptn*-Tg mice) and the specific role of RPTPβ/ζ (MY10 treatment) in the ethanol-induced neuroinflammatory process, cytokine levels were measured through multiplex luminometry in the PFC of adolescent mice 18 h after a single injection of ethanol (6 g/kg, i.p.) (Figure 1). An effect of the genotype for all the variables, together with an effect of the interaction sex x genotype for IL1B and TNFα was found (Table S1). No remarkable changes were observed in the protein levels of IL1β (Figure 1a,b) and TNFα (Figure 1g,h) after ethanol administration. However, acute ethanol tended to increase IL6 levels in the PFC of mice from all the experimental groups (Figure 1c,d). ANOVA revealed that treatment with ethanol significantly affected the PFC levels of IL6 in *Ptn*-Tg comparisons (Figure 1c and Table S1a) and in MY10-treated groups (Figure 1d and Table S1c). In addition, three-way ANOVA showed a significant effect of both genotype and treatment on MCP1 (Figure 1e) levels in PFC and a significant interaction between variables (Table S1a,b). An ethanol-induced increase in MCP1 in *Ptn*^+/+^ mice was prevented in *Ptn*-Tg mice (Figure 1e). Interestingly, this effect tends to be replicated in *Ptn*^+/+^ male mice when RPTP is blocked by MY10, but seems to be sex-dependent, as it was not observed in females (Figure 1f).

### 3.2. Ptn Overexpression Potentiates the Increases of Il6 and Ccl2 mRNA Levels in Female Mice and Blocks the Upregulation of Tnfa Expression in Males after Acute Ethanol Exposure

**Figure 2:**
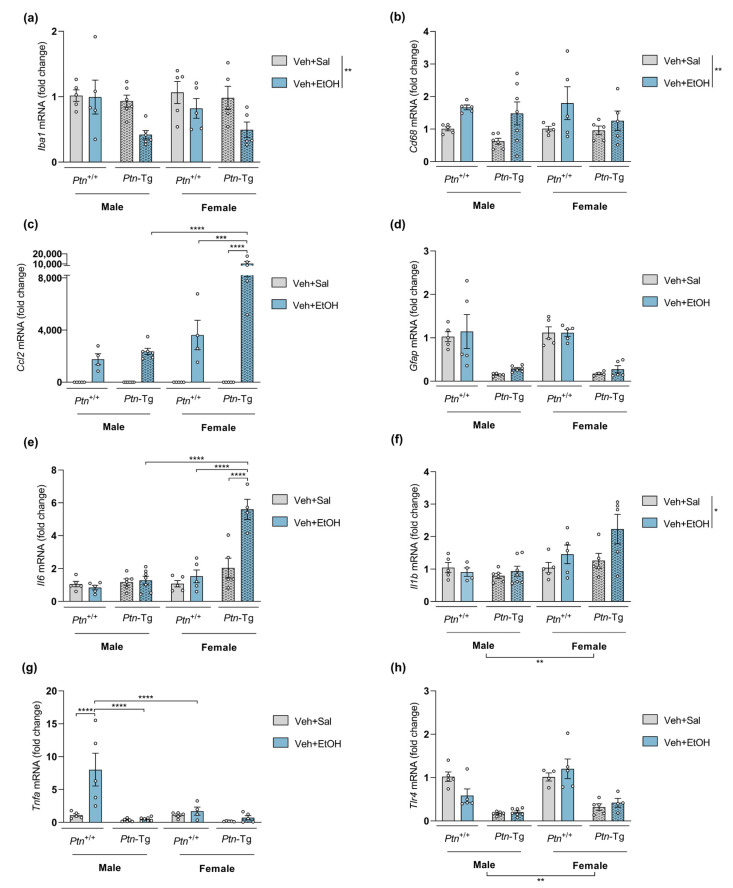
Effect of genetic overexpression of *Ptn* on ethanol-induced gene expression changes: (**a**) *Iba1* (ionized calcium-binding adapter molecule 1) mRNA; (**b**) *Cd68* (cluster of differentiation factor 68) mRNA; (**c**) *Ccl2* (C-C motif chemokine 2) mRNA; (**d**) *Gfap* (glial fibrillary acidic protein) mRNA; (**e**) *Il6* (Interleukin 6) mRNA; (**f**) *Il1b* (Interleukin 1 Beta) mRNA; (**g**) *Tnfa* (tumor necrosis factor alpha) mRNA; (**h**) *Tlr4* (toll-like receptor 4) in the PFC of adolescent *Ptn*^+/+^ and *Ptn*-Tg male and female mice 18 h after a single dose of ethanol (6 g/kg, i.p.). Three-way ANOVA analysis was performed. Data are presented as mean ± SEM (n = 5–6/group). * *p* < 0.05; ** *p* < 0.01; *** *p* < 0.001; **** *p* < 0.0001.

First, the effect of *Ptn* overexpression was studied by comparing the actions of ethanol on *Ptn*^+/+^ and *Ptn*-Tg adolescent mice (Figure 2). Three-way ANOVA revealed that ethanol treatment significantly affected mRNA levels of the microglia markers *Iba1* (Figure 2a) and *Cd68* (Figure 2b), as well as the proinflammatory marker *Il1b* (Figure 2f). However, ethanol did not modify *Gfap* levels of expression (Figure 2d), and only a significant effect of genotype was found. Detailed results of the statistical analysis are included in Table S2. In addition, the mRNA level of *Il1b* was significantly affected by sex. Adolescent female mice showed higher levels of *Il1b* than males (Figure 2f). A similar effect was found when *Tlr4* levels of expression were measured: significant differences were found regarding sex, genotype, and the interaction of both factors (Figure 2h and Table S2). We also found that prefrontal *Ccl2* (MCP1) and *Il6* levels are significantly increased in female *Ptn*-Tg mice after ethanol treatment when compared to ethanol-treated female *Ptn*^+/+^ mice and to ethanol-treated male *Ptn*-Tg mice (Figure 2c,e). Additionally, in male *Ptn*^+/+^ mice, *Tnfa* was found overexpressed after ethanol exposure, although this effect was fully prevented in *Ptn*-Tg mice. Very surprisingly, the levels of *Tnfa* mRNA were unaltered by ethanol administration or genotype in adolescent female mice (Figure 2g). To better understand these differences, correlations between the expression levels of all the factors and *Ptn* expression were analyzed in ethanol-treated *Ptn*^+/+^ and *Ptn*-Tg mice. Interestingly, *Ptn* and *Tnfa* mRNA levels significantly (*p* = 0.032) and positively correlate only in male *Ptn*^+/+^ receiving ethanol (Figure S1), which may contribute to the important difference in this experimental group compared with the others regarding *Tnfa* mRNA levels (Figure 2h). On the other side, a significant (*p* = 0.009) positive correlation was found between *Ptn* and *Gfap* mRNA levels in the PFC of adolescent female ethanol-treated *Ptn*^+/+^ mice (Figure S1), suggesting the participation of astrocytes in the sex differences observed after ethanol exposure.

**Figure 3:**
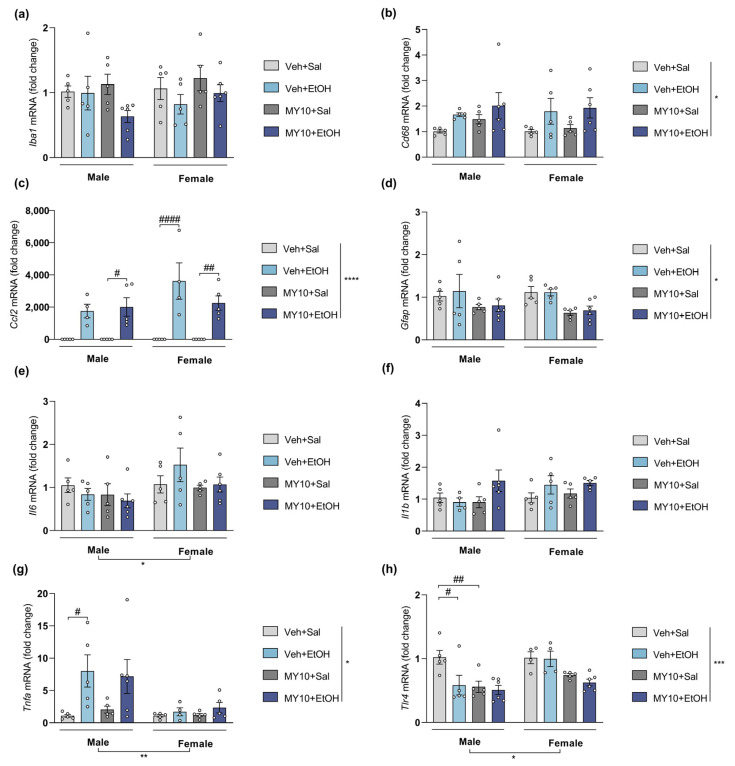
Effect of RPTPβ/ζ inhibition on ethanol-induced gene expression changes: (**a**) *Iba1* (ionized calcium-binding adapter molecule 1) mRNA; (**b**) *Cd68* (cluster of differentiation factor 68) mRNA; (**c**) *Ccl2* (C-C motif chemokine 2) mRNA; (**d**) *Gfap* (glial fibrillary acidic protein) mRNA; (**e**) *Il6* (Interleukin 6) mRNA; (**f**) *Il1b* (Interleukin 1 Beta) mRNA; (**g**) *Tnfa* (tumor necrosis factor alpha) mRNA; (**h**) *Tlr4* (toll-like receptor 4) in the PFC of adolescent *Ptn*^+/+^ male and female mice 18 h after treatment with a single dose of ethanol (6 g/kg, i.p.). One-hour prior, mice were administered vehicle (Veh) or MY10 (60 mg/kg, i.g.). Two-way ANOVA analysis was performed. Data are presented as mean ± SEM (n = 5–6/group). * *p* < 0.05; ** *p* < 0.01; *** *p* < 0.001 **** *p* < 0.0001. # *p* < 0.05, ## *p* < 0.01; #### *p* < 0.0001 for differences between treatments within the same sex.

We also evaluated whether MY10 administration modulates the expression of genes involved in neuroinflammation in the PFC of adolescent *Ptn*^+/+^ mice after a single injection of ethanol. We found that the pharmacological inhibition of RPTPβ/ζ by MY10 did not modulate the expression of these markers in the adolescent PFC after acute ethanol administration (Figure 3, statistical analysis in Table S3). It is interesting to note that *Ptn* overexpression decreased prefrontal levels of *Gfap* in male and female adolescent brains regardless of the treatment received (Figure 2d). Here, we show that the pharmacological inhibition of RPTPβ/ζ with MY10 mimics these PTN actions (Figure 3d), suggesting that the PTN/RPTPβ/ζ signaling pathway modulates mRNA expression of the astrocytic marker *Gfap* in the PFC during adolescence. 

### 3.3. Effect of Ptn Overexpression and Pharmacological Inhibition of RPTPβ/ζ with MY10 on the Expression of Neuroinflammatory Markers after LPS Administration

**Figure 4:**
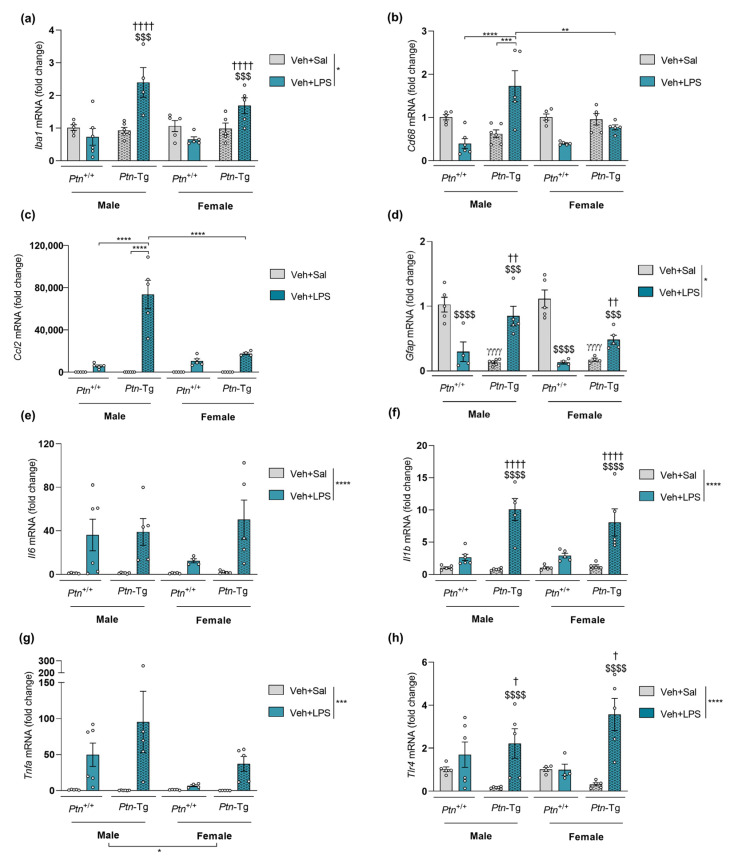
Effect of genetic overexpression of *Ptn* on LPS-induced gene expression changes: (**a**) *Iba1* (ionized calcium-binding adapter molecule 1) mRNA; (**b**) *Cd68* (cluster of differentiation factor 68) mRNA; (**c**) *Ccl2* (C-C motif chemokine 2) mRNA; (**d**) *Gfap* (glial fibrillary acidic protein) mRNA; (**e**) *Il6* (Interleukin 6) mRNA; (**f**) *Il1b* (Interleukin 1 Beta) mRNA; (**g**) *Tnfa* (tumor necrosis factor alpha); (**h**) *Tlr4* (toll-like receptor 4) mRNA levels in the PFC of adolescent *Ptn*^+/+^ and *Ptn*-Tg male and female mice 18 h after treatment with a single dose of LPS (5 mg/kg, i.p.). Three-way ANOVA analysis was performed. Data are presented as mean ± SEM (n = 5–6/group). * *p* < 0.05; ** *p* < 0.01, *** *p* < 0.001, **** *p* < 0.0001. If appropriate only genotype and treatment factors were considered. $$$ *p* < 0.001; $$$$ *p* < 0.0001 vs. vehicle-saline within the same genotype, excluding sex factor. † *p* < 0.05; †† *p* < 0.01; †††† *p* < 0.0001 vs. *Ptn*^+/+^ vehicle-LPS, excluding sex factor. γγγγ *p* < 0.0001 vs. vehicle-Sal, excluding sex factor.

To compare with ethanol effects, we studied in *Ptn*^+/+^ and *Ptn*-Tg adolescent mice the gene expression of the same neuroinflammatory markers after a general neuroinflammatory stimulus, LPS (Figure 4). Three-way ANOVA revealed that LPS treatment significantly affected mRNA levels of *Il6* (Figure 4e) and *Tnfa* (Figure 4g), with the latter also being affected by sex (the detailed statistical results are shown in Table S4a). However, *Ptn* overexpression did not seem to modify the levels of expression of these proinflammatory markers. LPS treatment and genotype significantly affected the mRNA levels of microglial marker *Iba1* (Figure 4a), astrocytic marker *Gfap* (Figure 4d)*,* cytokine *Il1b* (Figure 4e) and *Tlr4* (Figure 4h), and a significant interaction between variables was revealed (Table S4a, b). Moreover, *Ptn* overexpression induced a significant increase in mRNA levels of *Iba1* and *Il1b* in the PFC of LPS-treated mice in both sexes (Figure 4a,e). On the contrary, we found that only male *Ptn*-Tg mice treated with LPS showed significantly increased prefrontal levels of *Cd68* (Figure 4b) and *Ccl2* (Figure 4c). Specifically, genotype differences were found in *Cd68* mRNA levels analysis, while significant differences in *Ccl2* mRNA levels were found regarding treatment, sex, genotype, and the combinations of these variables (Table S4a). Interestingly, acute LPS exposure induced a decrease in *Gfap* levels in *Ptn*^+/+^ mice. However, in *Ptn*-Tg mice, significantly enhanced levels were found after LPS treatment (Figure 4d). It needs to be noted that basal levels (saline-treated mice) of this marker were found to be significantly diminished in *Ptn*-Tg mice.

**Figure 5:**
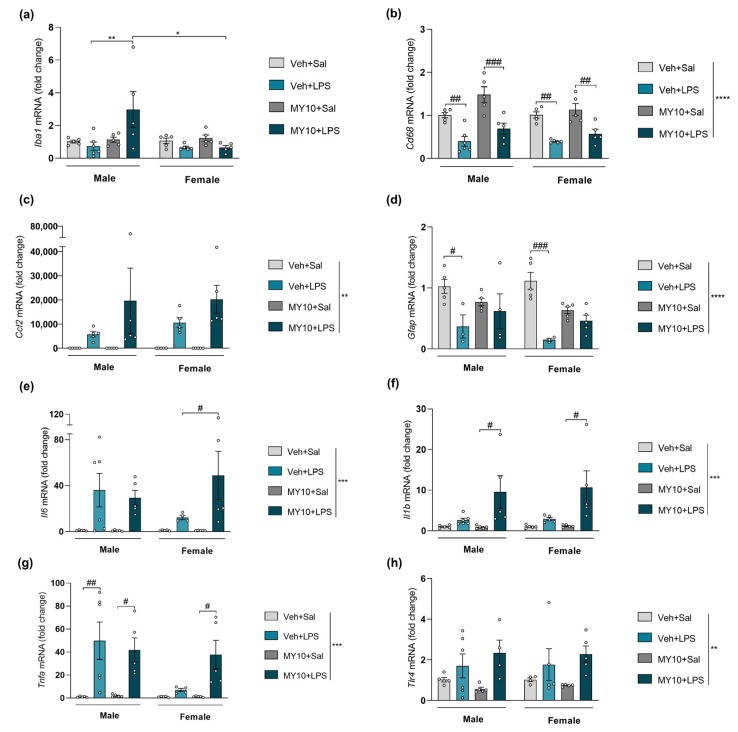
Effect of RPTPβ/ζ inhibition on LPS-induced gene expression changes: (**a**) *Iba1* (ionized calcium-binding adapter molecule 1) mRNA; (**b**) *Cd68* (cluster of differentiation factor 68) mRNA; (**c**) *Mcp1* (C-C motif chemokine 2) mRNA; (**d**) *Gfap* (glial fibrillary acidic protein) mRNA; (**e**) *Il6* (Interleukin 6) mRNA; (**f**) *Il1b* (Interleukin 1 Beta) mRNA; (**g**) *Tnfa* (tumor necrosis factor alpha) (**h**) *Tlr4* (toll-like receptor 4) mRNA in the PFC of adolescent *Ptn*^+/+^ male and female mice 18 h after treatment with a single dose of LPS (5 mg/kg, i.p.). One-hour prior, mice were administered vehicle (Veh) or MY10 (60 mg/kg, i.g.). Two-way ANOVA analysis was performed. Data are presented as mean ± SEM (n = 5–6/group). * *p* < 0.05; ** *p* < 0.01, *** *p* < 0.001, ****p < 0.0001; # *p* < 0.05, ## *p* < 0.01, ### *p* < 0.001 for differences between treatments within the same sex.

We also studied the involvement of RPTPβ/ζ in the neuroinflammation induced by LPS in the PFC of *Ptn*^+/+^ adolescent mice (Figure 5). The inhibition of RPTPβ/ζ by MY10 did not revert the changes in mRNA levels of *Cd68* (Figure 5b), *Ccl2* (Figure 5c), and *Tlr4* (Figure 5h) induced by LPS. Post-hoc comparisons revealed that LPS treatment only upregulates *Iba1* in males when the RPTPβ/ζ inhibitor, MY10, was present (Figure 5a). The decrease in *Gfap* mRNA levels observed in *Ptn*^+/+^ mice was absent when RPTPβ/ζ was inhibited by MY10 (Figure 5d). In addition, LPS only induced a significant upregulation of *Il1b* when RPTPβ/ζ was inhibited (Figure 5f) in a sex-independent manner. Interestingly, *Il1b* expression seems to follow the same tendency in this case as in *Ptn-*Tg (Figure 4f). Regarding changes in *Il6* (Figure 5e) and *Tnfa* (Figure 5g) levels, LPS treatment produced a milder effect in female mice. However, when RPTPβ/ζ is inhibited by MY10, the mRNA levels of *Il6* and *Tnfa* in the PFC of female mice are significantly increased to similar levels to the ones observed in LPS-treated males (for a detailed statistical analysis, see Table S5).

### 3.4. Comparative Study of PTN/RPTPβ/ζ Signaling Pathway after Ethanol and LPS Administration

**Figure 6:**
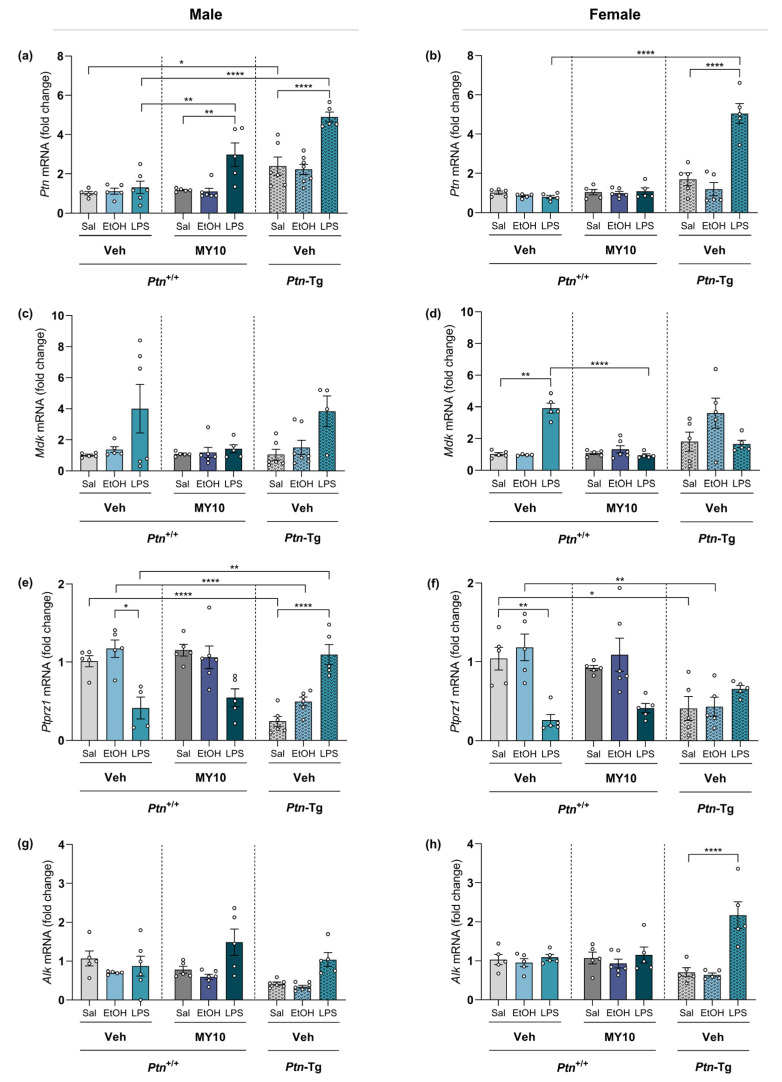
Modulation of PTN/MK/RPTPβζ signaling pathway after acute ethanol or LPS treatment. As the same control mice are used, each graph comparatively shows the effect of ethanol or LPS in two different sets: comparisons between *Ptn*^+/+^ and *Ptn*-Tg mice, and comparisons of *Ptn*^+/+^ mice treated with MY10 (or vehicle, Veh). *Ptn* (**a**,**b**), *Mdk* (**c**,**d**), *Ptprz1* (**e**,**f**), and *Alk* (**g**,**h**) mRNA levels are shown in the PFC of male (**left**) and female (**right**) adolescent mice. Data are presented as mean ± SEM (n = 3–7/group). * *p* < 0.05; ** *p* < 0.01; **** *p* < 0.0001.

To shed some light on the possible molecular mechanisms underlying ethanol and LPS-induced neuroinflammatory processes, we analyzed the expression of *Ptn*, *Mdk, Ptprz1,* and *Alk* in the PFC of adolescent mice after a single injection of ethanol (6 g/kg, i.p.) or LPS (5 mg/kg, i.p.) (Figure 6). *Ptn* levels were significantly increased after LPS acute exposure in *Ptn*-Tg mice of both sexes. Similarly, LPS induced the upregulation of *Ptn* mRNA levels in males treated with MY10. Interestingly, this upregulation was not observed in females (Figure 6a, b; statistical analysis in Table S6). In contrast, acute ethanol administration did not alter *Ptn* levels. We found that LPS induces an upregulation of *Mdk* levels in wild type mice, an effect that was prevented by treatment with MY10 (Figure 6c,d). As it happened with *Ptn* expression, ethanol treatment did not regulate *Mdk* expression. LPS reduced *Ptprz1* expression levels in wild type mice, an effect that was not apparently regulated by MY10 (Figure 6e,f). In addition, we found that *Ptn* overexpression led to a significant downregulation of *Ptprz1* in control (vehicle-saline) and ethanol-treated mice (Figure 6e,f). Interestingly, LPS prevented this downregulation of *Ptprz1* only in *Ptn*-Tg male mice (Figure 6e,f). We also measured mRNA levels of expression of *Alk*, a key downstream element of the RPTPβ/ζ pathway, to compare ethanol and LPS responses. Only an increase in *Alk* expression was found in LPS-treated female *Ptn*-Tg (Figure 6g,h). As ALK plays an important role in ethanol consumption [33] and could contribute to sex differences in the ethanol-induced response in the PFC [50], we aimed to explore the participation of Alk in these sex differences. To that, the group of mice included in ethanol experiments were also analyzed together, comparing males and females. In that case, an interesting effect of sex was found, both when *Ptn*-Tg mice were compared to *Ptn*^+/+^ and when the role of MY10 was studied in *Ptn*^+/+^ mice (Figure S2). Thus, ALK appears as a potential candidate to better understand sex differences in ethanol response. 

## 4. Discussion

Binge drinking constitutes a substantial risk for the adolescent immature brain. Ethanol-induced neuroinflammation during adolescence impairs PFC maturation and hippocampal neurogenesis and induces synaptic and myelin disarrangements [3,4,5,6]. Recently, we demonstrated that the hippocampal neurogenic loss induced by ethanol during adolescence was fully prevented when RPTPβ/ζ was inhibited by MY10 [44]. Glial morphological changes did not fully explain these actions of MY10 on the hippocampus or PFC; however, a possible modulation of neuroinflammation by RPTPβ/ζ in a sex-dependent manner cannot be ruled out. Thus, we aimed to shed some light on the role of the PTN/RPTPβ/ζ signaling pathway in the effects of ethanol related to neuroinflammation in the PFC, one of the more vulnerable regions in the adolescent brain [5,6], and to confirm if this participation is sex-affected. 

Ethanol-induced neuroinflammation is still controversial. Some hallmarks of the neuroinflammatory process are absent after ethanol consumption, while other reports strongly support the importance of neuroinflammation in ethanol actions [8,51]. Accordingly, we found that levels of IL1β and TNFα are not modified in PFC 18 h after acute ethanol, while this treatment significantly upregulates IL6 and MCP1 protein levels. Although neither *Ptn* overexpression nor RPTPβ/ζ inhibition by MY10 seems to alter the increase in IL6 levels induced by ethanol, the overexpression of the RPTPβ/ζ endogenous inhibitor PTN blocks the ethanol-induced increase in MCP1 levels (a summary of these effects on cytokine protein levels is presented in Table 2). Interestingly, this action of PTN overexpression does not seem to be uniquely mediated by RPTPβ/ζ since the treatment with MY10 does not alter MCP1 levels in response to ethanol in a significant manner. 

Ethanol treatment induced significant effects on *Iba1* and *Cd68* mRNA expression, which is consistent with results obtained by others using a different ethanol route of administration (intragastrical, i.g.) [18]. Thus, intraperitoneal ethanol administration replicates altered microglial gene expression induced by an acute i.g. ethanol dose, suggesting that ethanol-induced prefrontal microglial changes are independent from gut disruptions in adolescent mice. No significant effects of *Ptn* overexpression or RPTPβ/ζ pharmacological inhibition were detected on gene expression of microglial markers *Iba1* and *Cd68* (a summary of PTN and MY10 effects on gene expression is presented in Table 3). It is important to note that other authors have reported sex differences in microglial states in healthy brains and in pathological conditions, such as stress or neurodegeneration [52,53,54]. However, in our experimental conditions, ethanol effects on prefrontal *Iba1* mRNA levels are independent of sex. This correlates with the absence of sex differences in prefrontal Iba1 immunoreactivity after ethanol injury in adolescent mice [44].

The astrocytic response is proposed as a link between neuroinflammation processes induced by different stimuli. Here, we show that *Ptn* overexpression decreases prefrontal levels of *Gfap* in both sexes. The pharmacological inhibition of RPTPβ/ζ with MY10 mimics these PTN effects, suggesting an important role of astrocytes in the actions of the PTN/RPTPβ/ζ signaling pathway after ethanol exposure during adolescence. Interestingly, increased mRNA and protein level of GFAP has been reported only in female mice after ethanol drinking [9,50,55]. However, in our experimental conditions, *Gfap* prefrontal levels were not altered after ethanol administration. A limitation of this study is that gene expression is only measured at one timepoint, which may underlie this apparent discrepancy, together with other experimental conditions such as ethanol route of administration or the use of other astrocytic markers. The evidence supports the need to perform further studies to deeply dissect the participation of both PTN/RPTPβ/ζ cascade and astrocytic response in the acute ethanol-induced immune response and in the sex differences here identified.

Ethanol is known to increase the levels of proinflammatory cytokine TNFα via the participation of microglia [18,56]. Interestingly, this increment was fully prevented in ethanol-treated *Ptn*-Tg male mice, suggesting a role of PTN in the modulation of ethanol-induced changes in *Tnfa* expression. However, this effect is not replicated when RPTPβ/ζ is blocked by MY10, suggesting that this PTN action may be mediated by other receptors. Surprisingly, *Tnfa* levels were not altered in female mice after ethanol acute exposure and only a positive correlation with *Ptn* levels was found in males, evidencing different molecular mechanisms underlaying ethanol-induced neuroinflammation in the brain of female and male mice.

In contrast to the protein determinations performed, we found that the expression of *Il6* and *Ccl2* (MCP1) is significantly increased by ethanol in female *Ptn*-Tg mice, suggesting different actions of PTN in the PFC of adolescent male and female mice. Post-transcriptional modifications may be responsible for the differences in the levels of expression between protein and gene. It is worth noting that cytokines have short half-lives in both plasma and tissue [14].

On the other hand, *Il1b* expression does not seem to be significantly modulated by PTN or MY10. Ethanol withdrawal increases the expression of *Tnfa*, *Il1b,* and *Ccl2* whereas the expression of *Il6* is increased within the initial hours of acute intoxication [14,18,51,57]. However, *Il1b* prefrontal levels were not altered 18 h after intraperitoneal ethanol administration. It is important to note that this proinflammatory cytokine contributes to aberrant inflammation and has been found to be increased in the human alcoholic brain [51]. Furthermore, IL1β signaling is involved in the expression of other key elements of ethanol-induced neuroinflammation in the mouse brain such as *Il6*, *Ccl2,* and *Tnfa* [57,58]. Interestingly, microglia depletion does not counteract ethanol-induced increases of *Il1b*, *Il6,* and *Ccl2* [18], suggesting that other brain cell types may be the primary source of these proinflammatory markers. Neurons express *Ccl2* [59] and astrocytes have been shown to produce IL6 and IL1β and express *Ccl2* [14,18,60,61]. PTN could play an important role in these cells as *Ptn* upregulation modulates the expression of *Il6* and *Ccl2* and tend to increase *Il1b* prefrontal levels in adolescent females after acute ethanol treatment. PTN is known to be expressed in different cell types, including neurons and astrocytes [62], suggesting the actions of this neurotrophic factor in these cells may underlie the regulation of ethanol-induced changes in the levels of *Ccl2*, *Il6,* and *Il1b* in the PFC of *Ptn*-Tg mice. However, RPTPβ/ζ does not seem to be the main receptor mediating PTN actions because the inhibition of this receptor with MY10 does not significantly modulate ethanol-induced changes in the expression of these inflammatory markers.

Different studies support the role of the immune TLR4 response in ethanol-induced neuroinflammation and its detrimental effects in adult and adolescent mice [5,9,10,19,63]. Ethanol is known to contribute to gut leakiness and increase serum levels of LPS [56,64,65]. In fact, recent studies point to a connection between the release of LPS through systemic circulation and the neuroinflammation induced by ethanol [13,20,56]. In the present work, acute LPS exposure increases *Iba1* and *Cd68* mRNA levels in adolescent *Ptn*-Tg male mice, suggesting an enhancing effect of PTN on microglial responses. In addition, RPTPβ/ζ inhibition with MY10 potentiates *Iba1,* not *Cd68,* and mRNA levels increase after LPS, which is aligned with previous results obtained in adult mice exposed to LPS [42,43]. The data indicate an important role of the PTN/RPTPβ/ζ signaling pathway in LPS-induced overexpression of *Iba1* in the PFC and suggest *Ptn* overexpression effects on LPS-induced increase in *Cd68* levels are mediated through different PTN receptors. Very interestingly, these alterations were not replicated in female mice, indicating a sex-dependent contribution of PTN and RPTPβ/ζ to LPS-induced microglial responses. 

We observed LPS-induced increases in the levels of prefrontal expression of *Ccl2*, *Il6*, *Il1b,* and *Tnfa*, confirming previous studies by others [17,66]. Interestingly, *Ccl2* (MCP1) expression is dramatically increased in adolescent male mice exposed to LPS when *Ptn* is overexpressed. MCP1 is a key element in the activation of immune cells and production of inflammatory mediators in the brain during endotoxemia and can be produced by different cell types including microglia, astrocytes, and neurons [17,59,60]. Previous studies revealed that *Ptn* overexpression potentiates striatal astrocytosis and microglial activation after different stimuli, including LPS, in adult mice [27,43,67]. Another important factor involved in the effects of LPS, *Il1b* [66] is highly increased after LPS when *Ptn* is overexpressed or RPTPβ/ζ is inhibited with MY10 in both sexes. It is remarkable that the increase in *Il6* and *Tnfa* mRNA levels induced by LPS is less pronounced in females than males; however, this sex-dependent effect of LPS is counteracted when female mice are administered with MY10. Taken together, the data presented here show that the PTN/RPTPβ/ζ axis highly regulates LPS-induced neuroinflammation and suggests this pathway may be a target for the modulation of neuroinflammation in the adolescent brain. 

**Figure 7:**
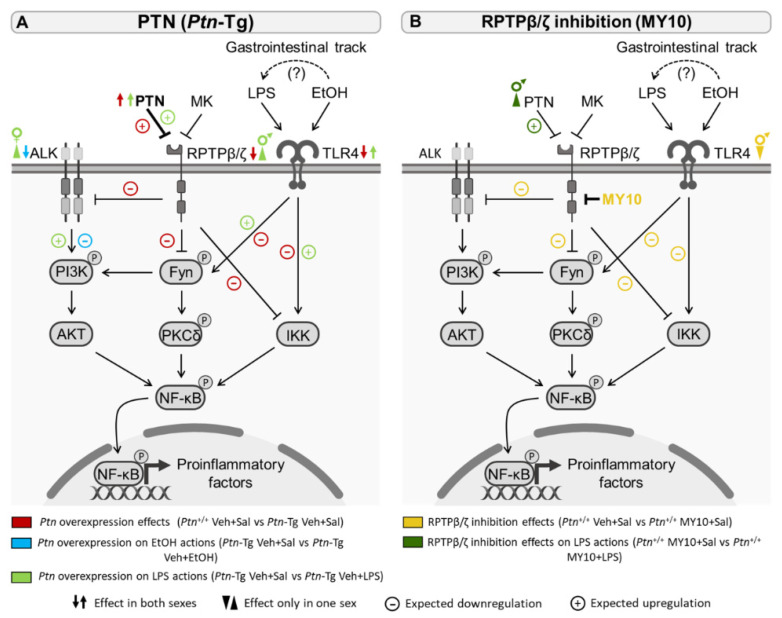
Proposed mechanisms of the effect of *Ptn* overexpression (**A**) and the pharmacological inhibition of RPTPβ/ζ with MY10 (**B**) on some of the principal signaling pathways implicated in ethanol and/or LPS acute response in adolescent mice. The different significant effects found are discernible by color; red: *Ptn* overexpression effects (*Ptn*^+/+^ Veh+Sal vs. *Ptn*-Tg Veh+Sal); light green: *Ptn* overexpression on LPS actions (*Ptn-*Tg Veh+Sal vs. *Ptn*-Tg Veh+LPS); blue: *Ptn* overexpression on EtOH actions (*Ptn*-Tg Veh+Sal vs. *Ptn*-Tg Veh+EtOH); yellow: RPTPβ/ζ inhibition effects (*Ptn*^+/+^ MY10+Sal vs. *Ptn*^+/+^ Veh+Sal), dark green: RPTPβ/ζ inhibition effects on LPS actions (*Ptn*^+/+^ MY10+Sal vs. *Ptn*^+/+^ MY10+LPS). Symbols are combined with the colors. ↑↓ for significant effect found in both sexes; ▲▼ for effect found only in one sex; 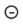 for expected downregulation and 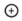 for expected upregulation.

Ethanol- and LPS-induced activation of TLR4 triggers intracellular signaling pathways that increase the transcription of different inflammatory cytokines and chemokines via NF-kB (Figure 7), including IL6, IL1β, and MCP1 [5,42,57,68]. TLR4 is expressed in microglia and other cell types, such as astrocytes. In fact, this receptor plays a crucial role in ethanol-induced immune response in astrocytes [18,68] and astrogliosis is detected in postmortem alcoholic human brains [69,70]. In addition, LPS induces astrocytosis in the mouse PFC [41,42], suggesting that the astrocytic response could be a link between neuroinflammatory processes induced by different stimuli. Moreover, it should be considered in the modulation of the levels of proinflammatory markers such as *Il6, Il1b,* and *Ccl2,* which cannot be explained by the microglial response [18,41,42].

Astrocytes were identified as MK-producing cells [71], and MK expression was increased in astrocytes in the prefrontal cortex of human alcoholics [26]. Ethanol exposure induced MK expression in neuronal cells, and it was found that MK contributed to the activation of ALK signaling in response to ethanol [72]. In the present work, ethanol exposure did not alter *Ptn, Mdk*, *Ptprz1,* or *Alk* expression in the PFC of adolescent mice. In contrast, *Mdk* levels were upregulated after LPS exposure, whereas the expression of its receptor *Ptprz1* was decreased, probably as a compensatory mechanism, like that observed in mice overexpressing the other endogenous ligand of this receptor, *Ptn*. Importantly, the administration of MY10 prevented an LPS-induced increase in *Mdk*. In contrast, pharmacological inhibition of RPTPβ/ζ with MY10 caused a significant upregulation of *Ptn* only in the PFC of male mice, whereas transgenic *Ptn* overexpression further potentiated the upregulation of *Ptn* expression in both males and females treated with LPS (Figure 7). These results are intriguing because the RPTPβ/ζ signaling pathway can be regulated both by MK and PTN, through the modulation of tyrosine phosphorylation of substrates of RPTPβ/ζ which, in turn, are known regulators of neuroinflammation. Activation through tyrosine phosphorylation of Fyn kinase, a substrate of RPTPβ/ζ, mediates LPS-induced activation of the NFκB pathway, suggesting that PTN/RPTPβ/ζ regulates proinflammatory cascades [27,37]. Accordingly, cerebral overexpression of *Ptn* potentiates LPS-induced microglial activation and neuroinflammation, whereas LPS-induced astrocytosis was blocked in the PFC of *Ptn*^−/−^ and *Mdk*^−/−^ mice [41,43]. It is necessary to note that the main findings of this manuscript are based only on changes in gene expression. It seems essential to study in detail possible differences in the expression of genes and proteins to fully understand the role of the PTN/RPTPβ/ζ signaling pathway in ethanol- and LPS-induced neuroinflammation.

## 5. Conclusions

Proinflammatory markers *Ccl2*, *Il6,* and *Il1b* seem to have an important role in the PTN modulation of ethanol effects during adolescence. Our data suggest that not all the PTN actions on the neuroinflammation induced by ethanol are mediated by RPTPβ/ζ. In addition, the contribution of PTN and RPTPβ/ζ to the changes in microglial gene marker expression after ethanol and LPS exposure during adolescence shows differences that strongly suggest the implication of more cell types in these PTN/RPTPβ/ζ effects. Remarkably, important sex differences regarding ethanol and LPS actions during adolescence, and their PTN/RPTPβ/ζ modulation, are reported. 

## Figures and Tables

**Table 1:** Primer sets used for qPCR analysis. *Alk: A*naplastic lymphoma kinase; *Ccl2*: Chemokine (C-C motif) ligand 2; *Cd68*: cluster of differentiation factor 68; *Gfap:* glial fibrillary acidic protein; *Iba1*: ionized calcium-binding adapter molecule 1; *Il1b*: Interleukin 1 beta; *Il6*: Interleukin 6; *Mdk*: Midkine; *Ptn*: Pleiotrophin; *Ptprz1*: Protein Tyrosine Phosphatase receptor type Z1; *Rpl37*: Ribosomal protein RPL37; *Tlr4:* toll-like receptor 4; *Tnfa:* tumor necrosis factor α.

Gene	Primer Forward	Primer Reverse
*Alk*	5′-TAAAGACGCTGCCAGAAGT-3′	5′-GGTGGTTGAATTTGCTGATG-3′
Ccl2	5′-GGCTCAGCCAGATGCAGTTAA-3′	5′-CCTACTCATTGGGATCATCTTGCT-3′
*Cd68*	5′-TGGCGGTGGAATACAATGTG-3′	5′-GATGAATTCTGCGCCATGAA-3′
*Gfap*	5′-AACAACCTGGCTGCGTAT-3′	5′-CTGCCTCGTATTGAGTGC-3′
*Iba1*	5′-GTCCTTGAAGCGAATGCTGG-3′	5′-CATTCTCAAGATGGCAGATC-3′
*Il1b*	5′-GCTGAAAGCTCTCCACCTCA-3′	5′-AGGCCACAGGTATTTTGTCG-3′
*Il6*	5′-TAGTCCTTCCTACCCCAATTTCC-3′	5′-TTGGTCCTTAGCCACTCCTTC-3′
*Mdk*	5′-TGATGGGAGCACTGGCAC-3′	5′-CATTGTACCGCGCCTTCTT-3′
*Ptn*	5′-TTGGGGAGAATGTGACCTCAATAC-3′	5′-GGCTTGGAGATGGTGACAGTTTTC-3′
*Ptprz1*	5′-CTACACAGGAGCACTAAATC-3′	5′-CTGTTTTCCCAGTGTTGTGA-3′
*Rpl37*	5′-ACCGCAGATTCAGACATGGATT-3′	5′-AGCGTAGGATCCCAGAGCAA-3′
*Tlr4*	5′-TAGGACTCTGATCATGGCACTG-3′	5′-GGAACTACCTCTATGCAGGGAT-3′
*Tnfa*	5′-AGGCACTCCCCCAAAAGATG-3′	5′-TGAGGGTCTGGGCCATAGAA-3′

**Table 2:** Overview of the effect of PTN genetic overexpression (*Ptn*-Tg) and RPTPβ/ζ inhibition (treatment with MY10) on protein level changes induced by ethanol. Data represented in Figure 1 are summarized. ns: no significant changes; (-): significant decrease; grey: both sexes.

Protein	Ethanol	
	PTN (*Ptn*-Tg)	RPTPβ/ζ Inhibition (MY10)
IL1β (Figure 1a,b)	ns	ns
IL6 (Figure 1c,d)	ns	ns
MCP1 (Figure 1e,f)	**(-)**	ns
TNFα (Figure 1g,h)	ns	ns

**Table 3:** Overview of the effect of PTN genetic overexpression (*Ptn*-Tg) and RPTPβ/ζ inhibition (treatment with MY10) on mRNA expression changes induced by ethanol (left) or LPS (right). Data represented in Figure 2, Figure 3, Figure 4 and Figure 5 are summarized. ns: no significant changes; (+): significant increase; (-): significant decrease; blue: males; orange: females; grey: both sexes.

mRNA Expression	Ethanol		LPS	
	PTN (*Ptn*-Tg)	RPTPβ/ζ Inhibition (MY10)	PTN (*Ptn*-Tg)	RPTPβ/ζ Inhibition (MY10)
*Iba1* (Figure 2, Figure 3, Figure 4 and Figure 5a)	ns	ns	**(+)**	(+)
*Cd68* (Figure 2, Figure 3, Figure 4 and Figure 5b)	ns	ns	**(+)**	ns
*Ccl2* (Figure 2, Figure 3, Figure 4 and Figure 5c)	**(+)**	ns	**(+)**	ns
*Gfap* (Figure 2, Figure 3, Figure 4 and Figure 5d)	ns	ns	**(+)**	ns
*Il6* (Figure 2, Figure 3, Figure 4 and Figure 5e)	**(+)**	ns	ns	**(+)**
*Il1b* (Figure 2, Figure 3, Figure 4 and Figure 5f)	ns	ns	**(+)**	**(+)**
*Tnfa* (Figure 2, Figure 3, Figure 4 and Figure 5g)	**(-)**	ns	ns	**(+)**
*Tlr4* (Figure 2, Figure 3, Figure 4 and Figure 5h)	ns	ns	**(+)**	ns

## Data Availability

The data presented in this study are available on reasonable request from the corresponding author.

## Supplementary Materials

The following supporting information can be downloaded at: https://www.mdpi.com/article/10.3390/biomedicines11051318/s1, Figure S1: Significant correlations are established between Tnfa, Gfap and Ptn mRNA level after ethanol acute exposure in the prefrontal cortex (PFC) of Ptn+/+ male mice and female mice; Figure S2: Effect of genetic overexpression of Ptn and RPTPβ/ζ inhibition on ethanol-induced Alk mRNA expression changes; Table S1: Statistical data of protein expression analysis after ethanol treatment; Table S2: Statistical data of mRNA expression analysis after ethanol treatment; Table S3: Statistical data of mRNA expression analysis after ethanol treatment; Table S4: Statistical data of mRNA expression analysis after LPS treatment; Table S5: Statistical data of mRNA expression analysis after LPS treatment; Table S6: Statistical data of mRNA expression analysis after ethanol or LPS treatment.
