# Supplementary material for: Implication of the PTN/RPTPβ/ζ Signaling Pathway in Acute Ethanol Neuroinflammation in Both Sexes: A Comparative Study with LPS
Source: Biomedicines. 2023 Apr 28;11(5):1318. doi: 10.3390/biomedicines11051318 (PMC10215719; doi:10.3390/biomedicines11051318)
Supplement: Supplementary file 1 [file biomedicines-11-01318-s001.zip › Figure S1_R1.pdf]

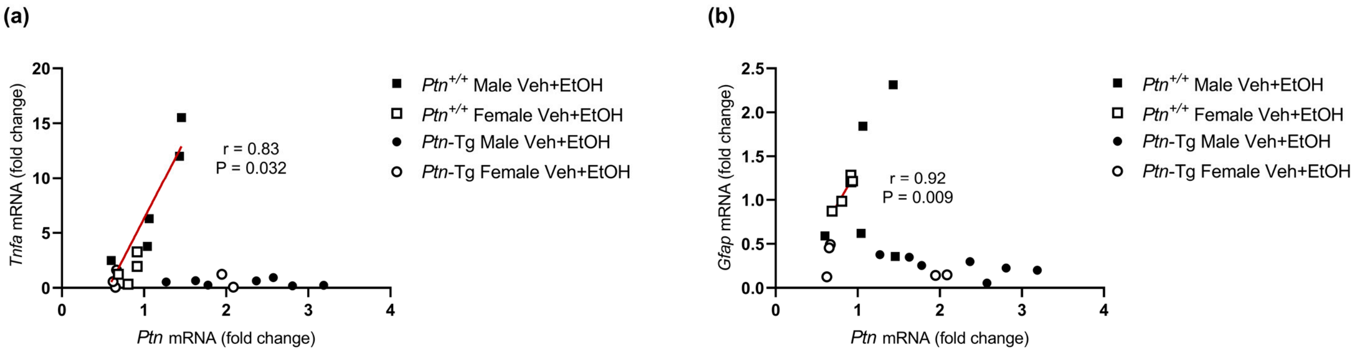

Figure S1. Significant correlations are established between *Tnfa* (**a**), *Gfap* (**b**) and *Ptn* mRNA level after ethanol acute exposure in the prefrontal cortex (PFC) of *Ptn*$^{+/+}$ male mice (**a**) and female mice (**b**). All groups were studied: *Ptn*$^{+/+}$ male and female mice and *Ptn*-Tg male and female mice. Correlations graphs shown pearson coefficients (r) and p values (P). Number of XY Pairs = 4-7/per group. Significant corresponding lineal regression is represented in red.
