# Supplementary material for: Implication of the PTN/RPTPβ/ζ Signaling Pathway in Acute Ethanol Neuroinflammation in Both Sexes: A Comparative Study with LPS
Source: Biomedicines. 2023 Apr 28;11(5):1318. doi: 10.3390/biomedicines11051318 (PMC10215719; doi:10.3390/biomedicines11051318)
Supplement: Supplementary file 1 [file biomedicines-11-01318-s001.zip › Figure S2_R1.pdf]

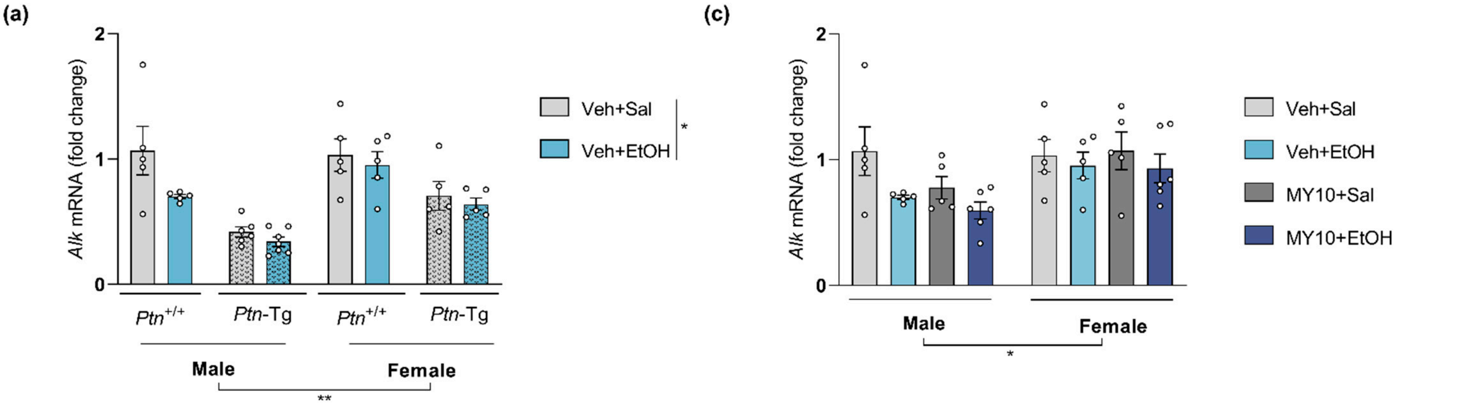

(b)

Treatment		Sex		Genotype	
Model	Sig.	Model	Sig.	Model	Sig.
$F_{1,35}$ = 4.66	p = .038	$F_{1,35}$ = 8.63	p = .006	$F_{1,35}$ = 36.37	p < .0001
**Treatment x Sex**		**Treatment x Genotype**		**Sex x Genotype**	
Model	Sig.	Model	Sig.	Model	Sig.
$F_{1,35}$ = 1.20	p = .282	$F_{1,35}$ = 1.22	p = .185	$F_{1,35}$ = 1.83	p = .185

Treatment x Sex x Genotype	
Model	Sig.
$F_{1,35}$ = 1.01	p = .322

(d)

Treatment		Sex		Interaction	
Model	Sig.	Model	Sig.	Model	Sig.
$F_{3,34}$ = 2.32	p = .092	$F_{1,34}$ = 6.49	p = .015	$F_{3,34}$ = 1.01	p = .399

Figure S2. Effect of genetic overexpression of *Ptn* and RPTPβ/ζ inhibition on ethanol-induced *Alk* mRNA expression changes. Graphs show data from a genetic *Ptn* overexpression model (*Ptn*-Tg vs. $Ptn^{+/+}$ mice; **a**) and data from $Ptn^{+/+}$ mice with RPTPβ/ζ inhibition by MY10 (**c**). Data are presented as mean ± SEM (n = 5 – 6/group). Tables show the corresponding statistical data; (**b**) Three-way ANOVA of data from $Ptn^{+/+}$ and *Ptn*-Tg mice of both sexes, treated with ethanol and (**d**) two-way ANOVA of data from male and female $Ptn^{+/+}$ treated with MY10 and ethanol.
