# Supplementary material for: Implication of the PTN/RPTPβ/ζ Signaling Pathway in Acute Ethanol Neuroinflammation in Both Sexes: A Comparative Study with LPS
Source: Biomedicines. 2023 Apr 28;11(5):1318. doi: 10.3390/biomedicines11051318 (PMC10215719; doi:10.3390/biomedicines11051318)
Supplement: Supplementary file 1 [file biomedicines-11-01318-s001.zip › Table S1.pdf]

Table S1. Statistical data of protein expression analysis after ethanol treatment (a) Three-way ANOVA of data from *Ptn*$^{+/+}$ and *Ptn*-Tg mice of both sexes, treated with ethanol. **(b)** Grouped data of MCP1 protein expression in *Ptn*$^{+/+}$ and *Ptn*-Tg mice, treated with ethanol. Two-way ANOVA of grouped data. **(c)** Two-way ANOVA of data from male and female *Ptn*$^{+/+}$ treated with MY10 and ethanol.

(a)

Measure (Fig. 1)	Treatment		Sex		Genotype	
	Model	Sig.	Model	Sig.	Model	Sig.
IL1β (a)	$F_{1,35} = .30$	$p = .586$	$F_{1,35} = .01$	$p = .901$	$F_{1,35} = 20.31$	$\mathbf{p < .0001}$
IL6 (c)	$F_{1,35} = 14.33$	$\mathbf{p = .001}$	$F_{1,35} = .02$	$p = .878$	$F_{1,35} = 15.84$	$\mathbf{p = .0003}$
MCP1 (e)	$F_{1,33} = 7.71$	$\mathbf{p = .009}$	$F_{1,33} = .04$	$p = .840$	$F_{1,33} = 7.64$	$\mathbf{p = .009}$
TNFα (g)	$F_{1,33} = .18$	$p = .674$	$F_{1,33} = 1.87$	$p = .180$	$F_{1,33} = 27.10$	$\mathbf{p < .0001}$

Measure (Fig. 1)	Treatment x Sex		Treatment x Genotype		Sex x Genotype	
	Model	Sig.	Model	Sig.	Model	Sig.
IL1β (a)	$F_{1,35} = .01$	$p = .919$	$F_{1,35} = .17$	$p = .680$	$F_{1,35} = 5.58$	$\mathbf{p = .024}$
IL6 (c)	$F_{1,35} = .14$	$p = .708$	$F_{1,35} = .66$	$p = .421$	$F_{1,35} = 1.76$	$p = .193$
MCP1 (e)	$F_{1,33} = .01$	$p = .938$	$F_{1,33} = 5.22$	$\mathbf{p = .029}$	$F_{1,33} = .30$	$p = .586$
TNFα (g)	$F_{1,35} = .001$	$p = .971$	$F_{1,35} = .790$	$p = .380$	$F_{1,35} = 10.54$	$\mathbf{p = .003}$

Measure (Fig. 1)	Treatment x Sex x Genotype	
	Model	Sig.
IL1β (a)	$F_{1,35} = .44$	$p = .510$
IL6 (c)	$F_{1,35} = .79$	$p = .379$
MCP1 (e)	$F_{1,33} = .11$	$p = .744$
TNFα (g)	$F_{1,35} = .72$	$p = .402$

(b)

Fold change	***Ptn*$^{+/+}$**		***Ptn*-Tg**	
	Sal	EtOH	Sal	EtOH
MCP1 (e)	18.694 ± 0.982	74.444 ± 17.498 $$	13.655 ± 1.306	19.817 ± 2.645 ††

Measure (Fig. 1)	Treatment		Genotype		Interaction	
	Model	Sig.	Model	Sig.	Model	Sig.
MCP1 (e)	$F_{1,37} = 8.66$	$\mathbf{p = 0.006}$	$F_{1,37} = 8.19$	$\mathbf{p = 0.007}$	$F_{1,37} = 5.54$	$\mathbf{p = 0.024}$

(c)

Measure (Fig. 1)	Treatment		Sex		Interaction	
	Model	Sig.	Model	Sig.	Model	Sig.
IL1β (b)	$F_{3,34} = .54$	$p = .659$	$F_{1,34} = .14$	$p = .710$	$F_{3,34} = 1.06$	$p = .380$
IL6 (d)	$F_{3,33} = 3.58$	$\mathbf{p = .024}$	$F_{1,33} = .53$	$p = .470$	$F_{3,33} = .94$	$p = .432$
MCP1 (f)	$F_{3,32} = 2.45$	$\mathbf{p = .081}$	$F_{1,32} = 1.76$	$p = .194$	$F_{3,32} = 1.49$	$p = .236$
TNFα (h)	$F_{3,34} = .35$	$p = .7859$	$F_{1,34} = .07$	$p = .794$	$F_{3,34} = 1.56$	$p = .218$
