# Supplementary material for: Implication of the PTN/RPTPβ/ζ Signaling Pathway in Acute Ethanol Neuroinflammation in Both Sexes: A Comparative Study with LPS
Source: Biomedicines. 2023 Apr 28;11(5):1318. doi: 10.3390/biomedicines11051318 (PMC10215719; doi:10.3390/biomedicines11051318)
Supplement: Supplementary file 1 [file biomedicines-11-01318-s001.zip › Table S2_R1.pdf]

Table S2. **Statistical data of mRNA expression analysis after ethanol treatment.** Three-way ANOVA of data from *Ptn*$^{+/+}$ and *Ptn*-Tg mice of both sexes, treated with ethanol.

Measure (Fig. 2)	Treatment		Sex		Genotype	
	Model	Sig.	Model	Sig.	Model	Sig.
Iba1 mRNA (a)	$F_{1,34} = 9.30$	**$p = .004$**	$F_{1,34} < .0001$	$p = .999$	$F_{1,34} = 6.62$	**$p = .015$**
Cd68 mRNA (b)	$F_{1,35} = 11.96$	**$p = .001$**	$F_{1,35} = .11$	$p = .743$	$F_{1,35} = 2.40$	$p = .130$
Ccl2 mRNA (c)	$F_{1,32} = 57.23$	**$p < .0001$**	$F_{1,32} = 16.81$	**$p = .0003$**	$F_{1,32} = 9.22$	**$p = .004$**
Gfap mRNA (d)	$F_{1,32} = .60$	$p = .443$	$F_{1,32} = .02$	$p = .877$	$F_{1,32} = 61.22$	**$p < .0001$**
Il6 mRNA (e)	$F_{1,34} = 16.79$	**$p = .0002$**	$F_{1,34} = 38.09$	**$p < .0001$**	$F_{1,34} = 34.62$	**$p < .0001$**
Il1b mRNA (f)	$F_{1,34} = 4.60$	**$p = .039$**	$F_{1,34} = 12.64$	**$p = .001$**	$F_{1,34} = 1.42$	$p = .242$
Tnfa mRNA (g)	$F_{1,34} = 11.20$	**$p = .002$**	$F_{1,34} = 6.40$	**$p = .016$**	$F_{1,34} = 17.06$	**$p = .0002$**
Tlr4 mRNA (h)	$F_{1,33} = .13$	$p = .722$	$F_{1,33} = 9.22$	**$p = .005$**	$F_{1,33} = 71.22$	**$p < .0001$**

Measure (Fig. 2)	Treatment x Sex		Treatment x Genotype		Sex x Genotype	
	Model	Sig.	Model	Sig.	Model	Sig.
Iba1 mRNA (a)	$F_{1,34} = .22$	$p = .644$	$F_{1,34} = 3.14$	$p = .085$	$F_{1,34} = .36$	$p = .554$
Cd68 mRNA (b)	$F_{1,35} = .33$	$p = .572$	$F_{1,35} = .15$	$p = .699$	$F_{1,35} < .001$	$p = .979$
Ccl2 mRNA (c)	$F_{1,32} = 16.81$	**$p < .001$**	$F_{1,32} = 9.22$	**$p = .004$**	$F_{1,32} = 6.41$	**$p = .017$**
Gfap mRNA (d)	$F_{1,32} = 0.12$	$p = 0.726$	$F_{1,32} = 0.07$	$p = 0.796$	$F_{1,32} = 0.01$	$p = 0.917$
Il6 mRNA (e)	$F_{1,34} = 18.63$	**$p = .0001$**	$F_{1,34} = 12.91$	**$p = .001$**	$F_{1,34} = 21.96$	**$p < .0001$**
Il1b mRNA (f)	$F_{1,34} = 4.52$	**$p = .041$**	$F_{1,34} = 1.72$	$p = .198$	$F_{1,34} = 3.54$	$p = .069$
Tnfa mRNA (g)	$F_{1,34} = 5.80$	**$p = .021$**	$F_{1,34} = 7.66$	**$p = .009$**	$F_{1,34} = 6.67$	**$p = .014$**
Tlr4 mRNA (h)	$F_{1,33} = 4.62$	**$p = .039$**	$F_{1,33} = 1.41$	$p = .244$	$F_{1,33} = 0.57$	$p = .091$

Measure (Fig. 2)	Treatment x Sex x Genotype	
	Model	Sig.
Iba1 mRNA (a)	$F_{1,34} = .36$	$p = .554$
Cd68 mRNA (b)	$F_{1,35} = .83$	$p = .368$
Ccl2 mRNA (c)	$F_{1,32} = 6.41$	**$p = .017$**
Gfap mRNA (d)	$F_{1,32} = 0.04$	$p = 0.849$
Il6 mRNA (e)	$F_{1,34} = 8.52$	**$p = .006$**
Il1b mRNA (f)	$F_{1,34} = 0.20$	$p = .658$
Tnfa mRNA (g)	$F_{1,34} = 7.34$	**$p = .011$**
Tlr4 mRNA (h)	$F_{1,33} = 3.02$	$p = .091$
