# Supplementary material for: Implication of the PTN/RPTPβ/ζ Signaling Pathway in Acute Ethanol Neuroinflammation in Both Sexes: A Comparative Study with LPS
Source: Biomedicines. 2023 Apr 28;11(5):1318. doi: 10.3390/biomedicines11051318 (PMC10215719; doi:10.3390/biomedicines11051318)
Supplement: Supplementary file 1 [file biomedicines-11-01318-s001.zip › Table S3_R1.pdf]

Table S3. Statistical data of mRNA expression analysis after ethanol treatment. Two-way ANOVA of data from male and female *Ptn*$^{+/+}$ treated with MY10 and ethanol.

Measure (Fig. 2)	Treatment		Sex		Interaction	
	Model	Sig.	Model	Sig.	Model	Sig.
Iba1 mRNA (a)	$F_{3, 34} = 2.01$	$p = .131$	$F_{1, 34} = .54$	$p = .470$	$F_{3, 34} = .98$	$p = .415$
Cd68 mRNA (b)	$F_{3, 34} = 3.53$	$\mathbf{p = .025}$	$F_{1, 34} = .10$	$p = .751$	$F_{3, 34} = .18$	$p = .906$
Ccl2 mRNA (c)	$F_{3, 30} = 19.62$	$\mathbf{p < .0001}$	$F_{1, 30} = 2.80$	$p = .105$	$F_{3, 30} = 1.82$	$p = .164$
Gfap mRNA (d)	$F_{3, 34} = 3.37$	$\mathbf{p = .029}$	$F_{1, 34} = .17$	$p = .681$	$F_{3, 34} = .19$	$p = .903$
Il6 mRNA (e)	$F_{3, 34} = 0.90$	$p = .451$	$F_{1, 34} = 4.41$	$\mathbf{p = .043}$	$F_{3, 34} = .92$	$p = .442$
Il1b mRNA (f)	$F_{3, 32} = 2.60$	$\mathbf{p = .070}$	$F_{1, 32} = 1.50$	$p = .230$	$F_{3, 32} = .83$	$p = .490$
Tnfa mRNA (g)	$F_{3, 32} = 3.57$	$\mathbf{p = .025}$	$F_{1, 32} = 7.94$	$\mathbf{p = .008}$	$F_{3, 32} = 2.02$	$p = .131$
Tlr4 mRNA (h)	$F_{3, 32} = 8.77$	$\mathbf{p = .0002}$	$F_{1, 32} = 6.98$	$\mathbf{p = .013}$	$F_{3, 32} = 1.61$	$p = .207$
