# Supplementary material for: Implication of the PTN/RPTPβ/ζ Signaling Pathway in Acute Ethanol Neuroinflammation in Both Sexes: A Comparative Study with LPS
Source: Biomedicines. 2023 Apr 28;11(5):1318. doi: 10.3390/biomedicines11051318 (PMC10215719; doi:10.3390/biomedicines11051318)
Supplement: Supplementary file 1 [file biomedicines-11-01318-s001.zip › Table S4_R1.pdf]

Table S4. Statistical data of mRNA expression analysis after LPS treatment. (a) Three-way ANOVA of data from $Ptn^{+/+}$ and *Ptn*-Tg mice of both sexes, treated with LPS. **(b)** Grouped data of *Iba1, Gfap, Il1b, Tlr4* mRNA expression in $Ptn^{+/+}$ and *Ptn*-Tg mice, treated with LPS. Two-way ANOVA of grouped data.

(a)

**Measure (Fig. 4)**	**Treatment**		**Sex**		**Genotype**	
	**Model**	**Sig.**	**Model**	**Sig.**	**Model**	**Sig.**
Iba1 mRNA (a)	$F_{1,33} = 6.29$	**$p = .017$**	$F_{1,33} = 1.37$	$p = .250$	$F_{1,33} = 18.19$	**$p < .001$**
Cd68 mRNA (b)	$F_{1,34} = .53$	$p = .472$	$F_{1,34} = 2.22$	$p = .146$	$F_{1,34} = 9.39$	**$p = .004$**
Ccl2 mRNA (c)	$F_{1,32} = 61.12$	**$p < .0001$**	$F_{1,32} = 13.80$	**$p < .001$**	$F_{1,32} = 29.67$	**$p < .0001$**
Gfap mRNA (d)	$F_{1,30} = 5.54$	**$p = .025$**	$F_{1,30} = 1.90$	$p = .178$	$F_{1,30} = 10.05$	**$p = .003$**
Il6 mRNA (e)	$F_{1,33} = 22.84$	**$p < .0001$**	$F_{1,33} = .18$	$p = .676$	$F_{1,33} = 2.28$	$p = .140$
Il1b mRNA (f)	$F_{1,34} = 53.10$	**$p < .0001$**	$F_{1,34} = .22$	$p = .640$	$F_{1,34} = 21.93$	**$p < .0001$**
Tnfa mRNA (g)	$F_{1,33} = 15.69$	**$p < .001$**	$F_{1,33} = 4.61$	**$p = .039$**	$F_{1,33} = 2.49$	$p = .124$
Tlr4 mRNA (h)	$F_{1,32} = 21.35$	**$p < .0001$**	$F_{1,32} = .37$	$p = .546$	$F_{1,32} = 1.44$	$p = .239$

**Measure (Fig. 4)**	**Treatment x Sex**		**Treatment x Genotype**		**Sex x Genotype**	
	**Model**	**Sig.**	**Model**	**Sig.**	**Model**	**Sig.**
Iba1 mRNA (a)	$F_{1,33} = 2.24$	$p = .144$	$F_{1,33} = 23.28$	**$p < .0001$**	$F_{1,33} = 1.15$	$p = .291$
Cd68 mRNA (b)	$F_{1,34} = 9.76$	**$p = .004$**	$F_{1,34} = 26.71$	**$p < .0001$**	$F_{1,34} = 2.31$	$p = .138$
Ccl2 mRNA (c)	$F_{1,32} = 13.80$	**$p < .001$**	$F_{1,32} = 29.67$	**$p < .0001$**	$F_{1,32} = 19.51$	**$p = .0001$**
Gfap mRNA (d)	$F_{1,30} = 5.12$	**$p = .031$**	$F_{1,30} = 87.87$	**$p < .0001$**	$F_{1,30} = .76$	$p = .391$
Il6 mRNA (e)	$F_{1,33} = .24$	$p = .630$	$F_{1,33} = 2.05$	$p = .161$	$F_{1,33} = 1.69$	$p = .205$
Il1b mRNA (f)	$F_{1,34} = 0.67$	$p = .419$	$F_{1,34} = 22.20$	**$p < .0001$**	$F_{1,34} = .47$	**$p = .050$**
Tnfa mRNA (g)	$F_{1,33} = 4.59$	**$p = .040$**	$F_{1,33} = 2.71$	$p = .109$	$F_{1,33} = .11$	$p = .747$
Tlr4 mRNA (h)	$F_{1,32} = .15$	$p = .700$	$F_{1,32} = 12.98$	**$p = .001$**	$F_{1,32} = 2.95$	$p = .096$

**Measure (Fig. 4)**	**Treatment x Sex x Genotype**	
	**Model**	**Sig.**
Iba1 mRNA (a)	$F_{1,33} = 1.15$	$p = 0.291$
Cd68 mRNA (b)	$F_{1,34} = 9.77$	**$p = .004$**
Ccl2 mRNA (c)	$F_{1,32} = 19.51$	**$p = .0001$**
Gfap mRNA (d)	$F_{1,30} = .26$	$p = .613$
Il6 mRNA (e)	$F_{1,33} = 1.54$	$p = .224$
Il1b mRNA (f)	$F_{1,34} = 1.06$	$p = .310$
Tnfa mRNA (g)	$F_{1,33} = 0.10$	$p = .752$
Tlr4 mRNA (h)	$F_{1,32} = 2.16$	$p = .151$

(b)

**Fold change**	***Ptn* +/+**		***Ptn*-Tg**	
	Sal	LPS	Sal	LPS
Iba1 mRNA (a)	1.040 ± 0.090	0.699 ± 0.138	0.955 ± 0.090	2.005 ± 0.258 †††† $$$
Gfap mRNA (d)	1.070 ± 0.086	0.215 ± 0.078 $$$$	0.152 ± 0.015 γγγγ	0.668 ± 0.099 †† $$$
Il1b mRNA (f)	1.043 ± 0.102	2.756 ± 0.300	1.002 ± 0.126	9.065 ± 1.324 †††† $$$$
Tlr4 mRNA (h)	1.018 ± 0.069	1.416 ± 0.373	0.237 ± 0.041	2.892 ± 0.530 † $$$$

**Measure (Fig. 4)**	**Treatment**		**Genotype**		**Interaction**	
	**Model**	**Sig.**	**Model**	**Sig.**	**Model**	**Sig.**
Iba1 mRNA (a)	$F_{1,37} = 5.58$	**$p = .023$**	$F_{1,37} = 16.49$	**$p < .001$**	$F_{1,37} = 21.42$	**$p < .0001$**
Gfap mRNA (d)	$F_{1,34} = 4.85$	**$p = .034$**	$F_{1,34} = 9.15$	**$p = .005$**	$F_{1,34} = 79.11$	**$p < .0001$**
Il1b mRNA (f)	$F_{1,38} = 55.84$	**$p < .0001$**	$F_{1,38} = 22.96$	**$p < .0001$**	$F_{1,38} = 23.56$	**$p < .0001$**
Tlr4 mRNA (h)	$F_{1,36} = 21.79$	**$p < .0001$**	$F_{1,36} = 1.13$	$p = .295$	$F_{1,36} = 11.91$	**$p = .001$**
