# Supplementary material for: Implication of the PTN/RPTPβ/ζ Signaling Pathway in Acute Ethanol Neuroinflammation in Both Sexes: A Comparative Study with LPS
Source: Biomedicines. 2023 Apr 28;11(5):1318. doi: 10.3390/biomedicines11051318 (PMC10215719; doi:10.3390/biomedicines11051318)
Supplement: Supplementary file 1 [file biomedicines-11-01318-s001.zip › Table S5_R1.pdf]

**Table S5. Statistical data of mRNA expression analysis after LPS treatment.** Two-way ANOVA of data from male and female *Ptn*$^{+/+}$ treated with MY10 and LPS.

| Measure (Fig. 5) | Treatment | | Sex | | Interaction | |
|---|---|---|---|---|---|---|
| | **Model** | **Sig.** | **Model** | **Sig.** | **Model** | **Sig.** |
| *Iba1* mRNA (a) | $F_{3,33} = 2.62$ | $p = .067$ | $F_{1,33} = 3.76$ | $p = .061$ | $F_{3,33} = 4.02$ | $\mathbf{p = .015}$ |
| *Cd68* mRNA (b) | $F_{3,33} = 25.68$ | $\mathbf{p < .0001}$ | $F_{1,33} = 2.11$ | $p = .156$ | $F_{3,33} = 1.08$ | $p = .370$ |
| *Ccl2* mRNA (c) | $F_{3,32} = 6.43$ | $\mathbf{p = .002}$ | $F_{1,32} = .13$ | $p = .718$ | $F_{3,32} = .10$ | $p = .096$ |
| *Gfap* mRNA (d) | $F_{3,27} = 11.67$ | $\mathbf{p < .0001}$ | $F_{1,27} = 1.18$ | $p = .288$ | $F_{3,27} = .51$ | $p = .676$ |
| *Il6* mRNA (e) | $F_{3,32} = 6.98$ | $\mathbf{p = .001}$ | $F_{1,32} = 0.02$ | $p = .889$ | $F_{3,32} = 1.54$ | $p = .223$ |
| *Il1b* mRNA (f) | $F_{3,33} = 9.42$ | $\mathbf{p = .0001}$ | $F_{1,33} = 0.09$ | $p = .768$ | $F_{3,33} = 0.03$ | $p = .099$ |
| *Tnfa* mRNA (g) | $F_{3,32} = 9.01$ | $\mathbf{p = .0002}$ | $F_{1,32} = 3.39$ | $p = .075$ | $F_{3,32} = 2.50$ | $p = .077$ |
| *Tlr4* mRNA (h) | $F_{3,31} = .02$ | $p = .994$ | $F_{1,31} = .02$ | $p = .894$ | $F_{3,31} = .02$ | $p = .994$ |
