# Supplementary material for: Implication of the PTN/RPTPβ/ζ Signaling Pathway in Acute Ethanol Neuroinflammation in Both Sexes: A Comparative Study with LPS
Source: Biomedicines. 2023 Apr 28;11(5):1318. doi: 10.3390/biomedicines11051318 (PMC10215719; doi:10.3390/biomedicines11051318)
Supplement: Supplementary file 1 [file biomedicines-11-01318-s001.zip › Table S6_R1.pdf]

Table S6. Statistical data of mRNA expression analysis after ethanol or LPS treatment. (a). Two-way ANOVA of data from male *Ptn*$^{+/+}$ and *Ptn*-Tg mice, treated with ethanol. **(b)** Two-way ANOVA of data from female *Ptn*$^{+/+}$ and *Ptn*-Tg mice, treated with ethanol. **(c)**. One-way ANOVA of data from male *Ptn*$^{+/+}$ treated with MY10 and ethanol. **(d)** One-way ANOVA of data from female *Ptn*$^{+/+}$ treated with MY10 and ethanol.

(a)

Measure (Fig. 6) Males	Treatment		Genotype		Interaction	
	Model	Sig.	Model	Sig.	Model	Sig.
Ptn mRNA (a)	$F_{2,28} = 14.83$	$\boldsymbol{p < .0001}$	$F_{1,28} = 68.94$	$\boldsymbol{p < .0001}$	$F_{2,28} = 10.19$	$\boldsymbol{p < .001}$
Mdk mRNA (c)	$F_{2,27} = 6.82$	$\boldsymbol{p = .004}$	$F_{1,27} < .0001$	$p = .999$	$F_{2,27} = .02$	$p = .984$
Ptprz1 mRNA (e)	$F_{2,26} = 1.82$	$p = .182$	$F_{1,26} = 11.68$	$\boldsymbol{p = .002}$	$F_{2,26} = 32.78$	$\boldsymbol{p < .0001}$
Alk mRNA (g)	$F_{2,28} = 4.32$	$\boldsymbol{p = .034}$	$F_{1,28} = 5.34$	$\boldsymbol{p = .028}$	$F_{2,28} = 3.81$	$\boldsymbol{p = .034}$

(b)

Measure (Fig. 6) Females	Treatment		Genotype		Interaction	
	Model	Sig.	Model	Sig.	Model	Sig.
Ptn mRNA (b)	$F_{2,24} = 25.13$	$\boldsymbol{p < .0001}$	$F_{1,24} = 56.44$	$\boldsymbol{p < .0001}$	$F_{2,24} = 28.61$	$\boldsymbol{p < .0001}$
Mdk mRNA (d)	$F_{2,23} = 3.92$	$\boldsymbol{p = .0344}$	$F_{1,23} = .87$	$p = .362$	$F_{2,23} = 11.73$	$\boldsymbol{p = .0003}$
Ptprz1 mRNA (f)	$F_{2,23} = 4.92$	$\boldsymbol{p = .017}$	$F_{1,23} = 8.67$	$\boldsymbol{p = .007}$	$F_{2,23} = 13.62$	$\boldsymbol{p = .0001}$
Alk mRNA (h)	$F_{2,24} = 15.35$	$\boldsymbol{p < .0001}$	$F_{1,24} = 1.19$	$p = .285$	$F_{2,24} = 11.91$	$\boldsymbol{p = .0003}$

(c)

Measure (Fig. 6) Males	Treatment	
	Model	Sig.
Ptn mRNA (a)	$F = 7.59$	$\boldsymbol{p = .0002}$
Mdk mRNA (c)	$F = 2.44$	$p = .061$
Ptprz1 mRNA (e)	$F = 4.05$	$\boldsymbol{p = .008}$
Alk mRNA (g)	$F = 2.63$	$\boldsymbol{p = .047}$

(d)

Measure (Fig. 6) Females	Treatment	
	Model	Sig.
Ptn mRNA (b)	$F = 1.04$	$p = .419$
Mdk mRNA (d)	$F = 41.29$	$\boldsymbol{p < .0001}$
Ptprz1 mRNA (f)	$F = 7.68$	$\boldsymbol{p = .0002}$
Alk mRNA (h)	$F = 2.96$	$\boldsymbol{p = .032}$
